# Perceptions and Experiences of Key Informants in Eye Health on the Implementation of Eye Care Health Promotion Interventions in South Africa

**DOI:** 10.3390/healthcare12222289

**Published:** 2024-11-16

**Authors:** Hlabje Carel Masemola, Olivia Baloyi, Zamadonda Nokuthula Xulu-Kasaba

**Affiliations:** 1 Department of Public Health Medicine, School of Nursing and Public Health, University of KwaZulu-Natal, Durban 4041, South Africa; baloyio@ukzn.ac.za; 2 Department of Optometry, College of Health Sciences, University of KwaZulu-Natal, Durban 4041, South Africa; xulukasabaz@ukzn.ac.za

**Keywords:** eye health, eye health professionals, health care provision, health promotion interventions

## Abstract

**Background:** Eye care health promotion interventions aim to encourage the adoption of healthy behaviours that impact eye health and vision impairment, as well as increase the use of eye care services. Thus, this study aims to explore and describe the perceptions of eye care coordinators on the implementation of eye care health promotion interventions in rural Limpopo Province. **Methods:** This exploratory, descriptive qualitative study employed individual in-depth interviews to collect data from 10 district eye health coordinators. Participants were purposely sampled between June and July 2024. Data were analysed thematically using NVivo version 12. **Results:** The study revealed seven key themes: human resources in eye health, resource management, policy and governance, eye care services, innovation in eye health, community and patient engagement, and coordination and referral systems. **Conclusions:** In summary, our study offers important insights into the challenges and opportunities in implementing eye care health promotion interventions. A recommendation is made to address identified challenges such as workforce shortages, inadequate infrastructure, fragmented policies and outdated technologies. Policymakers and eye health professionals can work towards achieving the goals of universal health coverage (UHC) in eye health, ultimately improving eye health outcomes.

## 1. Introduction

Eye health plays a vital role in overall health and well-being; however, it is frequently neglected in public health conversations. Vision impairments and eye diseases have significant impacts on individuals’ quality of life, productivity, and socioeconomic status. According to the World Health Organization (WHO), at least 2.2 billion people globally have a vision impairment or blindness, of which over 1 billion cases could have been prevented or are yet to be addressed through various interventions [1,2]. Eye health promotion interventions are strategies aimed at encouraging the adoption of healthy behaviours that impact ocular health and vision impairment while increasing the use of eye care services [2]. Eye care health promotion interventions are essential in addressing this issue as they encourage healthy behaviours that have an impact on eye health, increase the uptake of eye care services, and minimise preventable cases of visual impairment [3]. These interventions encompass a wide range of strategies such as public awareness campaigns, screening programmes, health education, policy advocacy, training programmes, community engagement, school-based programmes, and access to eye care services [3].

Integrating eye health promotion into hospitals and primary healthcare (PHC) settings is crucial for a comprehensive approach to healthcare, promoting the early detection, prevention, and management of eye conditions. In providing continuity of care, hospitals function in conjunction with PHC settings as essential access points for specialised eye care services as PHC settings serve as initial points of contact within the healthcare system, offering essential services [4]. This is consistent with the Primary Health Care Operational Model of the World Health Organization (WHO), which highlights the incorporation of basic health services, such as eye care, within PHC to attain universal health coverage and equitable health outcomes [5,6].

The Sustainable Development Goals (SDGs) underscore the importance of health for all, with Goal 3 aiming to ensure healthy lives and promote well-being for all at all ages [7]. Eye health promotion is crucial in achieving these goals by preventing avoidable blindness and visual impairment. Research conducted in Ghana and India demonstrates that the inclusion of primary eye health in community health services results in an increased utilisation of eye care facilities, better access to medications, and a preference for seeking professional care rather than using traditional remedies [8,9]. For instance, interventions focusing on increasing outdoor time for children show promising results in reducing the development and progression of myopia, a significant public health concern globally [10]. Furthermore, it has been demonstrated that cataract surgical interventions improve beneficiaries’ social inclusion, quality of life, and independence, highlighting the wider social benefits of addressing eye health issues [11]. However, there is limited research on the perceptions and experiences of those who implement these interventions, particularly in resource-limited settings like Limpopo Province in South Africa.

Global health outcomes have improved significantly as a result of effective eye health promotion initiatives. The World Health Organization’s Package of Eye Care Interventions has facilitated the development of evidence-based eye care interventions, highlighting the significance of evidence-based recommendations in promoting eye health and identifying critical, evidence-based interventions required for attaining universal eye health coverage [12]. Studies have demonstrated that systematic eye health initiatives can lead to the early detection and treatment of eye conditions, thereby reducing the prevalence of visual impairments and blindness. Research from a variety of places, including Morocco and sub-Saharan India’s Uttar Pradesh, highlight the significance of these initiatives [9,13,14]. Initiatives such as “VISION 2020: The right to sight”, led by the World Health Organization (WHO) and the International Agency for the Prevention of Blindness (IAPB), aimed to eliminate avoidable blindness globally by 2020 through strategic action plans and partnerships [15,16]. These international initiatives highlight how critical it is to comprehend and address local challenges that eye care coordinators face when putting these interventions into practice.

In Africa and around the world, fragmented policies that do not make specific mention of eye health promotion remain barriers to the implementation of initiatives aimed at promoting eye health [17,18]. Limited eye health services due to financial constraints, traditional treatments, and poor infrastructure, along with a need for better health literacy and governance in primary healthcare, remain significant challenges [17,19,20]. Additionally, a shortage of human resources, inadequate educational skills, limited funding, poor outreach, transportation difficulties, and fear of surgery impede the widespread implementation of comprehensive eye care models in low- and middle-income countries [21]. This underscores the need to strengthen health systems and leverage technological innovations to improve eye care services [21].

Despite the recognised importance of eye health and the introduction of various interventions aiming to reduce the burden of eye diseases, there is a lack of data on how these interventions are perceived by those at the forefront of eye health services. Key informants in the eye care sector play a crucial role in the success of these initiatives. Key informants such as district eye coordinators play a pivotal role in the success and bridging of these gaps in the implementation of eye care health promotion interventions. They are responsible for planning, implementing, and monitoring eye health promotion interventions. However, their insights on the effectiveness, challenges, and opportunities associated with these interventions remain underexplored. This study aims to explore and describe the perceptions and experience of eye care coordinators on the implementation of eye care health promotion interventions in Limpopo Province. By doing so, it seeks to identify barriers and facilitators in the current implementation strategies and provide insights for enhancing eye health services in the region.

## 2. Materials and Methods

### 2.1. Research Design

This was an exploratory convergent parallel mixed-method study. This article utilised an exploratory qualitative research design as part of a larger study. Exploratory qualitative research is an approach that aims to explore and understand the meaning of participants’ views of the situation under investigation [22]. This paper presents qualitative findings with the aim of exploring and describing the perceptions and experiences of eye care coordinators on the implementation of eye care health promotion interventions in Limpopo Province, South Africa.

### 2.2. Study Settings and Context

The study was conducted in Limpopo, a rural province of South Africa. Limpopo Province is landlocked in the northern part of the country, bordering Botswana, Zimbabwe, and Mozambique. It also borders the Mpumalanga, Gauteng, and North West provinces, with a total geographical area of approximately 125,754 square kilometres. The province has five district municipalities, namely Sekhukhune, Waterberg, Mopani, Capricorn, and Vhembe districts [23]. The province is categorised as rural by Statistics South Africa and has a population of 5.7 million, making it the fifth-largest province in the country [24]. The provision of eye health follows the District Health System (DHS) model in South Africa. In Limpopo, eye health district coordinators manage eye care services conducted in all clinics and hospitals—district, regional, and tertiary—within their jurisdiction. The district coordinators, in turn, report to the provincial eye health coordinator. The selected district coordinators were identified as relevant data sources as they oversee various districts’ optometry services across 37 hospitals in that province.

### 2.3. Population and Sampling

The target population in this study was ten (*n* = 10) eye health district coordinators from five districts in Limpopo Province. The participants were selected because they were coordinators in the districts, and senior optometrists overseeing the implementation of eye care health promotion interventions across all districts in the province. (see Table 1). A sampling strategy employed in this study is a non-probability sampling method where the researcher selects participants according to their judgement about who will be most informative [25]. We chose this method because eye health coordinators in the districts have experience in the implementation of eye care health promotion interventions and are employed by the Department of Health in Limpopo to provide an overseer of eye care services within their districts. Therefore, they were the relevant population to be purposively sampled due to their expertise, and we anticipated that they would provide rich and detailed insights regarding the health promotion interventions provided in each district.

### 2.4. Inclusion and Exclusion Criteria

Inclusion included all eye health district coordinators employed by the Department of Health in Limpopo and partly tasked with guiding outreach services in the districts. Participants voluntarily consented to participation in the study. Eye health professionals who were not eye health coordinators in the district, those on vacation or sick leave during the period of data collection, those not consenting to be part of the study, and those working in the private sector or academic institutions were excluded from the study.

### 2.5. Ethical Considerations

The Helsinki Declaration was followed in conducting the study taking into account voluntary participation, confidentiality during data collection, and the reporting of findings [26]. This study was conducted with ethical approval from the Biomedical Research Ethical Committee at the University of KwaZulu-Natal (BREC/00006067/2023) and the Limpopo Department of Health Ethics Committee (LP_2024-03-010). Additionally, approvals were sought from the heads of institutions at the selected healthcare facilities that took part in the study. The researcher obtained written informed consent from the participants prior to data collection. In compliance with the Protection of Personal Information Act (POPIA), the principal investigator obtained informed consent from the participants and gathered only essential personal information.

### 2.6. Data Collection

Semi-structured, face-to-face interviews were used to gather data from June to July 2024. An interview guide was used to collect data from the participants, and the interviews were recorded using a digital recorder. The main open-ended questions were “How would you describe the status of eye care in the province?” and “What are the policies and protocols guiding the implementation of eye care interventions in your district?”. The concept of health promotion interventions in eye care was clarified as effective public health initiatives and clinical interventions that met needs related to eye disorders and vision impairment including promotion, prevention, treatment, and rehabilitation. A question such as “Please describe your experiences/observations with the implementation of eye care outreach activities/interventions” was asked, followed by probing “What are the greatest highlights/successes with the implementation of outreach activities?” and “What are the challenges with the implementation of outreach activities?” to gain clarifications of the barriers and facilities of the activities currently implemented and to make sure that the entire interaction was understood thoroughly. The interview guide was piloted for credibility with the help of an expert in the field of optometry and public health. The pilot was conducted in May 2024 amongst two optometry coordinators in KwaZulu-Natal Province. The pilot study’s findings were not included in the main study as its purpose was to test the feasibility and clarity of the interview guide to ensure that it effectively addressed the research questions. Following the interviews, all authors collaborated to amend and confirm the interview guide. Although minor modifications were made to the wording and some questions based on feedback from the two pilot study participants, no questions were removed.

#### Recruitment of Participants and Interview Process

**Figure 1:**
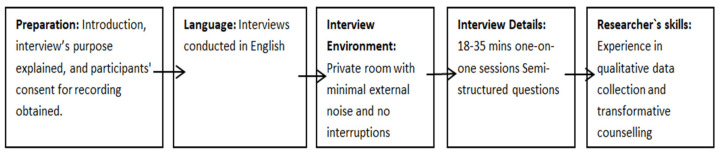
Flow chart representing the interview process.

Upon the receipt of ethical approval, the researcher arranged a meeting with the provincial coordinator to describe the study and explain its aims, objectives, and significance, inclusive of any ethical issues. Furthermore, during this meeting, the researcher allowed questions from the provincial coordinator to clarify any misconceptions. The researcher requested the provincial coordinator to share the details of the study with potential participants to enable willing participants to express interest and share their contact details with the researcher. Thereafter, the researcher sent all willing participants an information sheet and consent form to peruse through. The date and time of the face-to-face interviews were agreed upon with prospective participants. The contact details of the researcher, supervisor, and co-supervisor were made available for the participants for the communication of any concerns or questions. Each step of the interview process, from initial preparations to final execution details, including the researcher’s skills and the steps taken to ensure data quality and confidentiality, is captured in the flow chart (Figure 1).

### 2.7. Data Analysis

**Figure 2:**
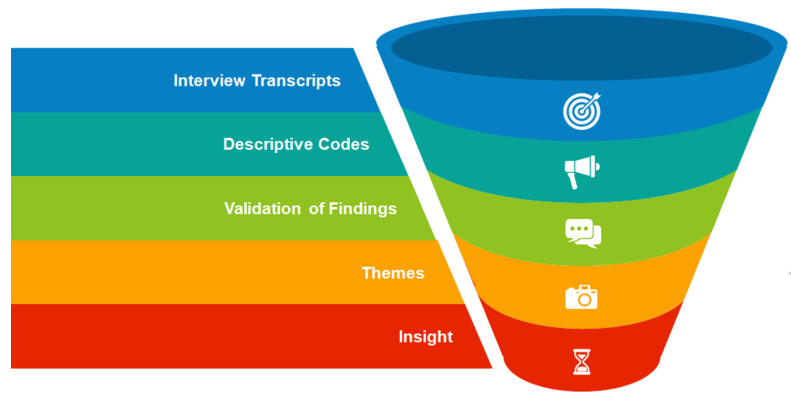
A tunnel diagram of the workflow for the process of the data analysis.

In this study, we employed Elo and Kyngäs’s (2008) content analysis approach, which involves a systematic process of preparation, organising of data, and reporting [27]. The interviews were audio-recorded, transcribed, and analysed in their original languages. Field notes confirmed that thematic saturation had been achieved [27]. During the preparation phase, we immersed ourselves in the data to gain a comprehensive understanding of the transcripts that were transcribed verbatim by a qualified language practitioner. In the organising phase, we conducted open descriptive coding, created categories, and developed higher-order categories. Finally, in the reporting phase, we described the content, presented the categories and subcategories, and used direct quotes to support our findings. This method ensured a thorough and structured analysis of the qualitative data. Nvivo version 12, a qualitative data software, was used in the coding of text data to present several categories until no new categories were generated. The principal investigator began by sharing the initial coding with a supervisor who acted as a peer reviewer to validate the codes. A manual codebook was then developed by the principal investigator and imported into NVivo V12. During the second phase of coding, the principal investigator organised and clarified similar descriptive codes to create themes, with supervisors confirming this process by performing inter-coder reliability checks to ensure the credibility and robustness of the analysis. Ultimately, the researcher formulated themes by grouping related codes from the second coding round and interpreting them into succinct, meaningful phrases to generate deeper insights and understand the underlying patterns and meanings within the data (Figure 2). Interview transcripts were filed orderly together with other documents collected during the interviews. All the collected information was saved in dated, named files.

### 2.8. Trustworthiness

Trustworthiness is one method by which researchers might convince readers and themselves that their research findings are important [28]. Lincoln and Guba (1985) improved the idea of trustworthiness by adding the criteria of credibility, transferability, dependability, and confirmability to mirror the conventional quantitative assessment criteria of validity and reliability [28,29]. The researcher had a prolonged engagement with participants to gain a deeper understanding of the experiences of participants, leading to richer and more nuanced data and also to build trust and rapport. Furthermore, before the main study began, a pilot study consisting of two interviews was conducted using the interview guide. In this study, the researcher provided a thick description by making findings such as behaviours and feelings more meaningful to others by describing them and their context in detail. Dependability was maintained by keeping an audit trail of the research steps taken from the start of a research project to the development and reporting of the findings. The researcher also continuously checked whether the data analysis process was in line with the standard of the chosen design. In this study, one supervisor acted as an adjunct coder and independently reviewed and confirmed the codes and themes identified by the principal investigator. This collaborative approach ensured that the findings were not influenced by the biases of a single researcher and validated the consistency and accuracy of the coding process. The involvement of an adjunct coder served as a form of triangulation, thereby strengthening the credibility and trustworthiness of the study’s conclusions. An audit trail of all the steps taken in the research path and their motives was kept for transparency.

## 3. Results

Ten coordinators for eye health who identified as female participated in our study. Participants were mostly in the 41–50 age range. When asked about their marital status, five said they were single, and five said they were married. They were all licensed optometrists with tertiary qualifications. The longest time spent working in the public sector was over 21 years, followed by a middle period of 15–20 years and a minimum period of 9–12 years.

Seven main themes and 12 sub-themes were identified regarding the perceptions and experiences of key informants in eye health on implementing eye care health promotion interventions. They are human resources in eye health, resource management, policy and governance, eye care services, innovation in eye health, community and patient engagement, and coordination and referral systems. The summary of the identified themes and subthemes is presented in Table 2 with supportive extracts referred to as participant number, gender, and rank.

Theme 1: Human resources in eye health
Sub-theme 1.1: Recruitment and retention

Participants noted significant challenges in recruiting skilled professionals, especially ophthalmic nurses, to support optometrists in the eye health field. This issue is attributed to the lack of training for ophthalmic nurses in the provinces; meanwhile, more ophthalmic nurses are leaving the profession for retirement. Participants pointed out a significant shortage of eye health professionals which becomes a primary challenge in the delivery of adequate eye health services. Some participants mentioned that often, due to a shortage of optometrists, they are not able to conduct community outreach as they have other responsibilities in their health facilities. Participants stressed that these recruitment and retention challenges have a direct impact on the quality and accessibility of eye care services, leading to gaps in service provision and overburdening the existing workforce.

*“Currently, the number of ophthalmic nurses is minimal because they are old and there is no more training for ophthalmic nurses…”*

(P1, F, AD)

*“Like the trend now it seems like ophthalmic nurses are decreasing in number.”*

(P2, F, AD)

*“…we are very short-staffed and so forth, so it is impossible that we can go and do extensive things for outreach.”*

(P10, F, SO)

Sub-theme 1.2: Workforce challenges

Participants raised a concern about skills gaps in terms of prescribing medication to patients and often rely on medical practitioners and ophthalmologists, and this in many cases results in a worsening of other eye conditions. Participants recognised the critical role of optometrists as decision-makers within the eye care workforce while also pointing out the challenges they face and the need for continued professional development and collaboration with other professionals. The majority of participants remain eager and motivated to remain in the public sector, highlighting a desire to have a meaningful impact on public health. A sense of purpose was also noted among participants to remain dedicated despite the challenges they faced. Participants further expressed concern about the significant backlogs in spectacles provision and eye operations, which are direct consequences of the eye care workforce’s inability to keep pace with the population’s needs. Participants proposed an urgent need for workforce expansion and better resource allocation in the eye health sector.

*“…treatment for eye drops, then you can recommend them to a general practitioner who will prescribe…because most hospitals don’t have optometrists with therapeutics.”*

(P1, F, AD)

*“…our district lacks ophthalmologists, so we have a cataract backlog. There are a lot of patients waiting for cataract removal and other stuff, like pterygium. So, it takes time for pathology patients to get help.”*

(P9, F, SO)

*“We don’t have enough staff to can do outreach, and if it was my wish it will be like at this moment we would have optometrists at the healthcare centres stationed there because we are not doing every clinic every day”*

(P5, F, AD)

*“…if each and every health facility had one or two professionals who specialise in eyes, it will make it easy.”*

(P4, F, CO)

Theme 2: Resource management
Sub-theme 2.1: Transportation

The participants expressed frustration about the lack of reliable motor vehicles to carry out school clinics, transport equipment, and efficiently reach communities in need. The quality and accessibility of eye care services are compromised by this transportation issue, especially in outreach settings, which also causes disruptions to their work. The successful delivery of healthcare services is dependent on logistics, as evidenced by the frequent references to damaged cars, vehicle unavailability, and the need for dedicated transportation.

*“The hospital cannot give two cars; that’s why we are saying it’s not really that it’s not doable, it’s just that it’s a challenge getting transport.”*

(P3, F, AD)

*“We were supposed to be doing school clinics, but we haven’t done that. It is because of the lack of resources, transport.”*

(P5, F, AD)

*“I wish that maybe they have like cars which are directly allocated for outreach services, so maybe it will improve the effectiveness of the transportation to the outreach”*

(P8, F, CO)

Sub-theme 2.2: Medical products

Participants stated how limited access to necessary medications and equipment at these clinics often forces patients to travel long distances to hospitals for treatment that could otherwise be managed locally. The lack of essential medications at the clinics, such as those for treating common conditions like conjunctivitis and glaucoma, results in patients being referred back to hospitals, creating a cycle of inefficiency and inconvenience for the patients. Participants further expressed concern about the limitations, particularly the inability to perform refractions or provide spectacles on-site, which forces patients to travel long distances to hospitals. The delays in receiving spectacles, backlog issues, and the seasonal nature of spectacle provision further exacerbate the problem, leading to patient dissatisfaction and inefficiencies in service delivery.

*“And also the issue of having availability of medication at the clinics; it’s very important also…”*

(P2, F, AD)

*“So, if the medication was supplied at their clinic closer to home, I think it would be better…”*

(P4, F, CO)

*“…the challenges we have regarding the spectacles, like providing spectacles, but with the other things I think everything is fine.”*

(P10, F, SO)

Sub-theme 2.3: Infrastructure and equipment

Participants frequently mentioned that the lack of adequate equipment hampers the effective delivery of specialised optometry services, particularly in areas like binocular-vision and low-vision care. This shortage extends to primary healthcare settings, where basic equipment is often missing, and forces professionals to carry their own or rely on limited resources. The absence of necessary equipment not only affects the quality of care but also leads to inefficiencies, such as over-referrals and under-referrals, because comprehensive testing cannot be performed on-site. Equipment is perceived as crucial to the success of eye health services where outdated or phased-out equipment currently in use further compromises the delivery of effective care. However, there is a sense of resilience and resourcefulness among participants to improvise with the limited resources available to keep their services ongoing.

*“We don’t have much of the speciality, the likes of binocular vision or low vision… especially in my hospital because we don’t have the equipment.”*

(P10, F, SO)

*“Our main issue will be lack of resources, lack of equipment…”*

(P5, F, AD)

*“I think we will do better if maybe we had mobile sort of equipment, handheld equipment…”*

(P2, F, AD)

The participants’ insights also reflect the impact of inadequate facilities on the quality of care and the ability to perform specialised optometry services effectively. Participants alluded to how the current infrastructure in many clinics does not meet the standards required for specialised optometry services. This shortfall compromises patient care as many clinics were designed to accommodate general medical doctors and nurses. There is insufficient space, inadequate lighting, and a lack of proper facilities, which restrict the ability to deliver comprehensive care within the required standards. During school outreach programs, a lack of proper facilities leads to challenges in false referrals, compromising the standard of care.

*“I would say the infrastructure—that the clinics are not designed for the specialised professions.”*

(P4, F, CO)

*“The infrastructure and everything, sometimes it’s affecting the quality of work that we should be doing. Sometimes the level of illumination itself and the busyness sometimes of the clinic, so I cannot say the quality are up to standard, but at least we are rendering the bare minimum service that we should render.”*

(P8, F, CO)

Sub-theme 2.4: Funding

Financial resources for critical services are insufficient for eye health because it appears that funding for eye care is either inadequate or not prioritised in the national healthcare budget. It is challenging to satisfy the expectations of patients and eye health professionals due to this lack of concentration. One major barrier to equipping hospitals and clinics with the necessary infrastructure and equipment is the limited money. The inability to provide outreach services is another issue raised by participants regarding the budgetary deficit. They think that increasing funding allocated especially for eye health could significantly improve the quality of care provided.

*“So it’s… budget, that’s one of the problems. We don’t have all the equipment”*

(P1, F, AD)

*“I think the only suggestion, unfortunately, it involves the money. If they can at least give us the provision, especially the budget, I think that is where the problem lies.”*

(P10, F, SO)

*“…the problem is the financial backup regarding the outreach, hence we are not performing that much.”*

(P10, F, SO)

Theme 3: Policy and governance
Sub-theme 3.1: Policy development and implementation

Several participants are aware of the protocols and policies in place that guide their work. These include screening protocols, comprehensive eye test protocols, and policies to organise services in the hospitals or on outreach activities. Several participants also seemed to imply some kind of review and redesign phase based on learnings from past experiences, which implies a commitment towards service delivery improvement. While some participants indicated that the policies are still being developed, a substantial amount of progress has been achieved with formal structures and working papers existing at national and provincial levels.

*“Yes, we do have those protocols. We have screening protocol; we have comprehensive eye test protocol…”*

(P1, F, AD)

*“I would say maybe they are working for us because usually the protocols it’s us that review them, I mean, from the past experiences we sit down and check if our protocols are still serving us or where can we improve and all that.”*

(P2, F, AD)

Several participants also commented on the fragmented nature of policies, where they need to sometimes create their protocols oftentimes at provincial or hospital levels because there is no central national policy. Despite such policies, a few participants suggested that these policies are not always put into practice as they should be, which results in the service delivery system collapsing back to the lowest quality. It is significant to note that many areas like health promotion in eye care have no specific policies or guideline, which makes it ambiguous for the professionals working there. Participants also highlighted how policies may not be completely implemented even when they are in place due to inadequate funding and resources, including personnel and equipment.

*“We had to develop some of our protocols based on what is it that is required to be done when you go outside or when you do comprehensive eye clinic setup.”*

(P1, F, AD)

*“There is a national policy, it is still a draft, and we have our own policy drafted from national; then from the policy we have protocols.”*

(P3, F, AD)

*“Our wish is to do health promotion at the clinics. But like I said, that is why when I talk about the policy it is where we have constraints.”*

(P5, F, AD)

Theme 4: Eye care services
Sub-theme 4.1: Accessibility and availability

Many participants noted that optometry services are available in almost all hospitals, with optometrists regularly visiting clinics through outreach programs. This structure ensures that patients, even in remote areas, have access to eye care. Participants highlighted the effectiveness of outreach programs, where optometrists visit clinics on scheduled days to provide screenings and other services. Participants indicated that community knowledge concerning the location-based provision of different eye care services is generally high, the need for spectacles being commonly understood and known resources for these services identified well. While some participants gave services a high rating for availability and accessibility, others took a more cautious “50/50” approach, stating that while services are generally available, there is still room for improvement in terms of reaching all patients and ensuring prompt care.

*“Then we have 37 hospitals that have got optometry services, with optometrists working in those hospitals.”*

(P3, F, AD)

*“And also the issue of availability, that they already know that optometry services would be at clinic A on such a day, because we give programmes to clinics that they put out.”*

(P3, F, AD)

*“On a scale of 1 to 10, I would say we are at 7 because we have optometrists in almost all the hospitals, so optometry services are rendered, and we do outreach services to the primary healthcare facilities.”*

(P1, F, AD)

*“Overall, I would say it’s 50/50 because our patients are aware that we have different eye clinics or optometry sections in different hospitals.”*

(P4, F, CO)

Sub-theme 4.2: Affordability

The beneficial effects of outreach initiatives and locally offered eye care services were emphasised by participants as ways to address accessibility and affordability. Participants observe that patients save money on transportation and other expenditures by having services brought directly to local clinics; this makes healthcare more inexpensive, particularly in a province where a large number of people live in poverty. Participants brought up the issue of the clinic’s effective and free services, which allow patients to receive care without having to travel far. Participants noted that since patients are saving time and money by not having to go far, they are satisfied with the local services. Participants thought outreach initiatives are effective as well, especially when it comes to helping patients manage their eye problems locally and prevent expensive hospital trips, which lessens their financial burden.

*“Our province, you know, 80% is indigent, meaning that 80% cannot afford moving around, so taking services to them is indirectly addressing the issue of affordability because they don’t have to travel long distances.”*

(P3, F, AD)

*“There is no need for them to travel; we are saving them money to come to the hospitals.”*

(P5, F, AD)

*“I think the outreach, for me, is really, really working well for the patients because it is also reducing their costs, in terms of transportation, and then moreover, at the clinic also, the services are free.”*

(P8, F, CO)

Theme 5: Innovation in eye health
Sub-theme 5.1: Technology and Treatment Advancement

Participants voiced worries about advancements in treatment and technologies related to eye health. Participants acknowledged the pace of technical developments; however, certain institutions are falling behind due to old equipment. The necessity to adapt and grow to keep up with these developments was acknowledged by the participants. Participants drew attention to the use of extremely old VA Charts in clinics and raised concerns about the validity of the data produced using such old instruments. To enhance the standard of eye care services and stay up to date with continuous innovations in the area, participants also emphasised the critical need to update and invest in new technology.

*“Technologically things are changing and you find that some of us are still having old equipment and we need to evolve, but then we have to be somewhere in order for us to be where we want to be.”*

(P2, F, AD)

*“So, clinics are using very old VA Charts, very old that sometimes you would even question the results…”*

(P3, F, AD)

*“Things are evolving. There are hospitals that don’t have any kind of advanced equipment—they are still using basic—and already now technology has changed…”*

(P1, F, AD)

Theme 6: Community and patient engagement
Sub-theme 6.1: Health promotion

The significance of spreading awareness through various platforms, including social media, community radio stations, and health lectures, was emphasised by the participants. The public is intended to be informed about these services and encouraged to seek assistance through these efforts. Participants included conducting health talks, giving out consent forms in schools, and getting students involved in outreach to the community. This practical method guarantees that individuals in need, particularly in remote or underprivileged locations, receive eye care treatments. The use of both traditional (radio, pamphlets) and modern (social media) platforms for health promotion was highlighted. While some participants observed that conventional media is becoming less effective, others found benefits in combining these methods with more recent platforms, such as social media, which enable community interaction. Participants provided detailed descriptions of certain initiatives, frequently tied to awareness months, where eye care professionals actively engage with the community.

*“We also involve our community radio stations where we are able to answer a lot of questions and also make people aware of services.”*

(P3, F, AD)

*“Normally during the awareness month, it is where we go to the communities and give health promotion, health talks.”*

(P5, F, AD)

*“So, we are in social media time. Pamphlets and radio stations are no longer active because people no longer listen.”*

(P3, F, AD)

*“Also with the health talks that we also give at the clinics to educate them in terms of the relationships between other things and the eye, other conditions, chronic and all those things”*

(P2, F, AD)

Theme 7: Coordination and referral systems
Sub-theme 7.1: Referral pathways

The referral system that was emphasised by the participants is primarily defined by the collaborative relationship that exists between ophthalmologists and optometrists. Identifying eye diseases and referring patients to ophthalmologists is the primary responsibility of optometrists, especially for cataract operations and other pathologies. They said that following treatment, patients are frequently referred back to optometrists, suggesting a continuous process of inter-referrals that supports comprehensive care. The participants expressed a cautious attitude towards referrals, intending to avoid needless referrals while guaranteeing that all conditions receive appropriate examination and treatment. Maintaining this balance is essential to delivering comprehensive care without overloading the referral system.

Participants mentioned coordinating minor ailments on-site alongside primary healthcare providers, like nurses, as opposed to referring patients for easy-to-treat treatments, like eye drops. The majority of participants observed a hierarchical referral system in which clinics refer patients for comprehensive optometry services to district hospitals, and district hospitals refer more complex cases—like those requiring ophthalmology services—to higher-level regional or tertiary hospitals. There was also mention of referrals involving other healthcare system disciplines outside of optometry and ophthalmology, indicating a multidisciplinary approach to patient care.

*“Optometrists are the ones who mainly refer the patients to the ophthalmologists, and ophthalmologists make sure that they do the cataracts. They concentrate on surgeries and pathologies and refer back to us. It is inter-referral, we work well…”*

(P1, F, AD)

*“We usually just do the basic things there to identify our patients and refer them…we at least coordinate with the PHC or the nurses to come up with medication so that we don’t find ourselves referring a patient from X for a mere eye drop here.”*

(P2, F, AD)

*“We are using a referral system that is up and down. A district hospital gives support to clinics, then a regional hospital gives support to the district, then tertiary gives support to regional.”*

(P3, F, AD)

## 4. Discussion

In this study, the use of Nvivo 12 was crucial for organising and analysing qualitative data, as it facilitated a systematic approach to identifying and coding relevant themes and sub-themes from participant responses. Our inclusion and exclusion criteria, which focused specifically on eye health coordinators actively involved in implementing health promotion interventions, were designed to ensure that the data reflected the experiences and perspectives most appropriate to our research question. By selecting participants with direct involvement in these interventions, the analysis yielded themes and sub-themes that captured specific challenges and facilitators related to health promotion implementation.

The findings of this study noted significant challenges in recruiting skilled professionals, especially ophthalmic nurses, to support optometrists in the eye health field posing a significant challenge in the eye health sector. This result is consistent with the WHO’s “health workforce” building block, which highlights the necessity of an efficient health workforce that can adapt to changing population demands. Similar studies reveal that an even greater factor contributing to the scarcity of qualified eye health professionals is the lack of training, especially in rural areas [30]. The shortage of eye health professionals, particularly in rural areas, is a major barrier to achieving UHC in eye health. This challenge is not unique to South Africa, as is confirmed by Majid, H et al., who concluded that the shortage of training for ophthalmic nurses in various provinces remains a barrier to delivering effective services [17]. Participants stressed that these recruitment and retention challenges have a direct impact on the quality and accessibility of eye care services, leading to gaps in service provision and overburdening the existing workforce. Our findings are consistent with Christian et al., who underscore that community outreach initiatives are hampered by the lack of optometrists since those that are available are overworked in hospitals [31]. In the end, this condition affects patient care and results by creating gaps in the supply of services.

The study’s participants also noted the skills gap among optometrists not trained in therapeutics, particularly in prescribing medications, which often necessitates a reliance on medical practitioners in the absence of ophthalmologists. This finding points to the need for continuous professional development (CPD) and the enhancement of collaborative practices within the eye care workforce. Our results support the notion of a study conducted by Hansraj et al. that requirements for the ocular therapeutics certification of optometrists should reviewed to create an enabling environment for the acquisition of these skills [32]. The Eye Care Competency Framework (ECCF) places a strong emphasis on inter-professional cooperation to fill in optometrists’ skill gaps and assist multidisciplinary teams in providing complete eye care, which is essential for enhancing service delivery in settings with limited resources [33]. The desire among participants to remain in the public sector despite challenges reflects a strong sense of purpose, which is crucial for sustaining motivation and improving service delivery.

The lack of reliable transportation was identified as a major barrier to the delivery of eye care services, particularly in outreach settings. Our findings reaffirm a study in Kumasi Metropolis, Ghana which identified barriers like distance affecting eye care service utilisation [34]. Inadequate transportation not only limits the reach of eye care services but also exacerbates health inequities, particularly in rural and underserved areas. Other studies have outlined that inadequate transportation leads to delays in access to care, and participants in our study proposed a coordinated approach that involves improving infrastructure, allocating dedicated vehicles for eye care services, and enhancing logistical support for outreach programs [35,36,37]. The World Report on Vision emphasises that to make eye care a fundamental component of universal health coverage, it is still imperative to ensure that these services are available to everyone, even those who live in remote areas [2].

The study participants expressed concerns about the limited access to essential medication and the inadequacy of infrastructure in clinics which significantly hampers patient care often, necessitating long-distance travel to hospitals for treatments that could be managed at the clinic. This inefficiency is exacerbated by the lack of medication for common eye conditions and the inability to provide necessary services, such as comprehensive refractions and spectacles, on-site. Our findings are consistent with those of Khan et al., who found that clinics often lack essential medications for conditions like conjunctivitis and glaucoma, forcing patients to seek care at hospitals [38]. The study further highlighted that patients face significant delays in receiving spectacles, contributing to dissatisfaction and inefficiencies in service delivery [38]. Notwithstanding the overall shortage of resources in the larger health system, the scarcity of essential medications and spectacles not only hampers the delivery of effective eye care but also contributes to inefficiencies, such as unnecessary referrals and delays in treatment.

The issue of inadequate funding for eye health was a recurrent theme in the study, with participants highlighting the need for increased financial resources to support the expansion and improvement of eye care services. Our findings reaffirm the insufficiency of financial resources for eye health services which significantly compromise the quality of care and accessibility in various regions [39]. This finding underscores the importance of the WHO building block of “health system financing”, which calls for sustainable financing mechanisms to ensure the availability of essential health services, including eye care [40]. Our data support a notion discussed in several studies such as Christian et al.’s that many eye care facilities, particularly in low-income areas, lack essential resources to provide eye care services in primary healthcare settings [31]. This issue is compounded by inadequate prioritisation in national healthcare budgets, leading to a lack of infrastructure and equipment for outreach services.

Participants in the study expressed concerns about the fragmented nature of eye care policies and the challenges associated with their implementation. The lack of central national policies for eye health, as reported by participants, mirrors the situation in many low- and middle-income countries (LMICs), where eye care is often not integrated into national health policies [41,42]. This is also confirmed by a study conducted by Sithole which highlighted that fragmented eye care policies in South Africa remain, echoing global challenges [18]. This lack of central policies aligns with the WHO’s emphasis on integrating eye care into national health systems for effective governance. Our findings are similar to many regions in which the implementation of eye health policies faces multifaceted challenges stemming from infrastructural and systemic barriers [43,44]. The WHO Eye Care Competency Framework addresses concerns about fragmented eye care policies by emphasising comprehensive policies aligned with health system strategies for effective governance and leadership [33].

The study’s findings on the accessibility and availability of eye care services reflect the WHO building block of “service delivery” as many participants noted that optometry services are available in almost all hospitals and accessible through outreach programmes despite gaps in reaching all patients, particularly in remote areas [40]. This finding underscores the need for strengthening the delivery of eye care services to ensure that they are accessible to all, regardless of geographic location. Our study findings emphasise the use of outreach initiatives to improve access to eye care services which are supported by various studies reporting high community awareness regarding available eye care services, particularly the need for spectacles, as well as the effectiveness of these programmes in improving screening rates and facilitating timely interventions [45,46]. By contrast, many individuals face long distances to clinics and high costs associated with eye care, leading to an underutilisation of services, as seen in Ghana’s Kumasi Metropolis [34]. Another study in Kakamega municipality, Kenya, highlighted that refractive services are hindered by poor accessibility in government hospitals and affordability issues, impacting the uptake of eye care services [38]. The WHO “World Report on Vision” highlights the importance of integrating eye care into PHC services to improve accessibility and reduce disparities in eye health [2]. The report also emphasises the role of outreach programmes in extending eye care services to underserved populations, which aligns with the study’s findings on the effectiveness of outreach initiatives in Limpopo Province.

Participants in the study highlighted the role of outreach programmes and locally provided eye care services in improving the affordability of care. This finding is related to the Lancet Global Health Commission on Global Eye Health which emphasises the importance of making eye care services affordable and accessible to all, particularly in LMICs [47]. The integration of eye care into PHC services, as recommended by the WHO and other global health initiatives, can help reduce the financial burden on patients by bringing services closer to communities and minimising the need for costly hospital visits. Other studies noted substantial savings on transportation from outreach clinics eliminating the need for long-distance travel, making healthcare more affordable and accessible [48]. Therefore, outreach initiatives need to be aligned with local needs for their sustainability and long-term empowerment of communities.

The study’s findings on the use of outdated equipment in eye care facilities highlight the challenges associated with keeping pace with technological advancements in eye health. The rapid advancement of artificial intelligence (AI) and digital health technologies presents both opportunities and challenges for the global eye health sector. While AI and digital health have the potential to revolutionise the diagnosis and treatment of eye diseases, the study’s findings suggest that many eye care facilities in Limpopo Province are not equipped to fully leverage these technologies. Even though our study findings do not include the use of AI, evidence suggests that AI and telehealth can bridge gaps in eye care, especially in underserved regions, by providing remote consultations and screenings [49,50].

The study’s findings on health promotion activities in eye care highlight the importance of engaging communities and raising awareness about eye health services. This finding aligns with the World Report on Vision, which highlights the need for innovative approaches to health promotion, including the use of digital platforms and social media, to reach a wider audience. The study’s findings suggest that a combination of traditional and modern health promotion strategies can be effective in raising awareness and improving access to eye care services. According to Ouchene et al., community radio and pamphlets remain vital for disseminating health information, especially in remote areas where internet access may be limited [51]. Platforms like Facebook and X (formerly known as Twitter) are known to facilitate real-time interaction and engagement, allowing health professionals to connect with the community and share vital information quickly [52]. Some of the study participants provided evidence in support of this. Community engagement strategies such as health talks and health awareness were identified as major strategies used in eye health promotion. Our findings support a notion discussed in several studies such as Kasturia et al. and Jeyaraman et al. which emphasises the importance of utilising various platforms like social media and community radio for health promotion and engaging the public through informative initiatives [53,54].

The study’s findings on referral systems in eye care emphasise the importance of coordination and collaboration between different levels of care. Participants describe the referral system, which highlights the importance of collaboration between optometrists and ophthalmologists in delivering comprehensive eye care. Optometrists generally demonstrate higher referral accuracy compared to general practitioners, with studies indicating an 18.6% improvement in accuracy [55]. However, false-positive referrals remain a significant issue, contributing to the oversubscription of hospital eye services [55]. This inter-professional relationship is essential for giving patients swift and proper care, particularly in the case of eye diseases such as cataracts needing surgical intervention. As the custodians of primary eye care providers, optometrists can diagnose conditions and safely manage patients or refer them when intervention by an ophthalmologist is necessary, which keeps pathologies from going undetected while also minimising unnecessary referrals. An efficient referral system that matches patient needs with system capacity whilst only referring the cases requiring advanced care to higher-level facilities is essential.

Participants emphasised the need not to burden specialised services with unnecessary referrals. This is consistent with the overall aims of healthcare systems, which focus not solely on maximising limited resources but also on managing how patients flow through the system [56]. The ongoing referral process and flow of patients between optometrists and ophthalmologists is clear evidence of a collaborative effort to deliver complete care throughout the range of eye health services. Participants preferred to manage minor diseases on-site and in coordination with the primary healthcare professionals such as nurses instead of referring patients for basic treatments (e.g., eye drops). This strategy not only reduces the burden on the referral system but also enables local healthcare providers to treat minor or less severe eye conditions. Our findings support evidence that community eye clinics have shown a 27.5% reduction in first-visit referrals to specialist clinics, indicating the effective management of stable conditions at the community level [4]. The integration of nurses into the eye care process reinforces the significance of a multidisciplinary approach to care delivery.

The referral described by participants is designed hierarchically, where clinics refer patients to district hospitals for comprehensive optometry services and district hospitals in turn refer more complex cases to the regional or tertiary hospital for ophthalmology services. Such a tiered system ensures that the right level of care is provided to the patient regarding the complexity of the condition, further enhancing efficiency within the healthcare setting. Further, participants identified the involvement of other health professions in the referral process for patients, reflecting a more holistic and integrated service model necessary to best address patient health needs well beyond the scope of both optometry and ophthalmology. Indeed, this facilitation of services would prove invaluable in facilitating the best possible outcomes for patients with systemic conditions affecting eye health, such as diabetes and hypertension. The referral system described by the participants underscores the importance of collaboration, careful resource management, and a multidisciplinary approach to providing comprehensive eye care. This is further supported by the hierarchical referral process, whereby the level of complexity of a patient’s condition determines the level of care they are referred to which ultimately strengthens the overall health system and improves patient outcome [55].

## 5. Limitations

There were some limitations to the study. Participants knew they were being interviewed by an eye health professional, which could have introduced bias. However, the principal investigator ensured that the purpose of the research was clearly explained to all participants before data collection. Another limitation of this study is that all participants in the sample were female. This homogeneity may restrict the generalisability of our findings to the broader population, as it limits potential differences in perception and experiences among male or non-binary individuals. Additionally, the study employed non-probability sampling, which limits the ability to determine how well the sample represents the broader population. As a result, the generalisability of the findings beyond the study population may be limited.

## 6. Recommendation

To enhance eye health promotion interventions in Limpopo Province, we suggest a comprehensive strategy. First, enhancing human resource capacity through targeted recruitment, retention strategies, and continuous professional development will ensure a skilled workforce ready to effectively implement health promotion interventions. It is also vital to tackle resource management by improving infrastructure, securing necessary medical supplies, and strengthening logistical support for outreach programs. These actions will increase the quality of eye care services, especially in remote areas. Adequate funding is essential; increased investment in eye health will facilitate sustainable promotion efforts, allowing for consistent service delivery and community involvement. To address policy fragmentation, eye health should be incorporated into national health policies and budgets, creating a structured framework that aligns with broader health system objectives and resources. This includes enhancing outreach programs and integrating eye care into primary health care (PHC) services, which can improve accessibility and affordability for patients. Lastly, health promotion initiatives should embrace innovative, community-centred approaches that effectively raise awareness and promote preventive practices.

## 7. Conclusions

Perceptions of key informants in this study revealed a significant shortage of skilled eye health professionals, which directly hampers comprehensive eye care service delivery. Additionally, inadequate funding for eye health services remains a major barrier to the expansion and improvement of eye care. Policy and governance were flagged as key in the successful implementation of eye health promotion interventions. Fragmented eye care policies, and associated challenges, were found to retard eye health promotion implementation. The study findings underscore the importance of strengthening the delivery of eye care services to ensure they are accessible to all, regardless of geographic location. Health promotion activities emerged as pivotal in raising awareness about eye health services, highlighting the need for innovative approaches to engage communities and encouraging their utilisation of eye care services.

Overall, our study provides valuable insights into the challenges and opportunities in implementing eye care health promotion interventions in a rural setting like Limpopo Province. The findings align with global health initiatives, including the World Health Organization’s building blocks of a health system, and amplify the need for continued efforts to strengthen health systems and improve access to eye care services in South Africa.

## Figures and Tables

**Table 1:** Participant’s characteristics.

Participant No.	Age Group	Sex of the Participants	Marital Status	Highest Qualification	Years in the Public Sector	Rank *
**P1**	41–50	Female	Married	Masters of Optometry	21>	Assistant Director
**P2**	41–50	Female	Married	Masters of Optometry	15–20	Assistant Director
**P3**	51–60	Female	Married	Bachelor of Optometry	21>	Assistant Director
**P4**	41–50	Female	Single	Post-Graduate Diploma	15–20	Chief Optometrist
**P5**	41–50	Female	Single	Post-Graduate Diploma	21>	Assistant Director
**P6**	41–50	Female	Single	Bachelor of Optometry	11–15	Senior Optometrist
**P7**	41–50	Female	Single	Bachelor of Optometry	15–20	Senior Optometrist
**P8**	41–50	Female	Married	Bachelor of Optometry	21>	Chief Optometrist
**P9**	31–40	Female	Single	Bachelor of Optometry	15–20	Senior Optometrist
**P10**	31–40	Female	Married	Bachelor of Optometry	6–10	Senior Optometrist

Assistant Director (AD); Chief Optometrist (CF); Senior Optometrist (SO), * Ranking of job profile by the Department of Health.

**Table 2:** Summary of the identified themes and sub-themes.

Theme	Sub-Theme
**1. Human resources in eye health**	1.1. Recruitment and retention
	1.2. Workforce challenges
**2. Resource management**	2.1. Transportation
	2.2. Medical products
	2.3. Infrastructure and equipment
	2.4. Funding
**3. Policy and governance**	3.1. Policy development and implementation
**4. Eye care services**	4.1. Accessibility and availability
	4.2. Affordability
**5. Innovation in eye health**	5.1. Technology and treatment advancement
**6. Community and patient engagement**	6.1. Health promotion
**7. Coordination and referral systems**	7.1. Referral pathways

## Data Availability

Owing to the protection and privacy of participants, data from this qualitative study is the property of the University of KwaZulu-Natal and may be made available upon request from the Biomedical Research Ethical Committee at the University of KwaZulu-Natal or the study authors.
